# Multifunctional Elastic Nanocomposites with Extremely Low Concentrations of Single-Walled Carbon Nanotubes

**DOI:** 10.1021/acsami.2c01086

**Published:** 2022-04-14

**Authors:** Ilya V. Novikov, Dmitry V. Krasnikov, Anton M. Vorobei, Yaroslav I. Zuev, Hassaan A. Butt, Fedor S. Fedorov, Sergey A. Gusev, Alexander A. Safonov, Eugene V. Shulga, Stepan D. Konev, Ivan V. Sergeichev, Sergey S. Zhukov, Tanja Kallio, Boris P. Gorshunov, Olga O. Parenago, Albert G. Nasibulin

**Affiliations:** † Skolkovo Institute of Science and Technology, Nobel 3, 121205, Moscow, Russia; ‡ Aalto University School of Chemical Engineering, Kemistintie 1, 02015, Espoo, Finland; § Kurnakov Institute of General and Inorganic Chemistry of RAS, Leninskiy Prospekt 31, 119991, Moscow, Russia; ∥ Moscow Institute of Physics and Technology, Dolgoprudnyi, 141700, Moscow Region Russia

**Keywords:** single-walled carbon nanotubes, nanocomposite, thermoplastic polyurethane, coagulation precipitation, percolation threshold, piezoresistivity, EMI shielding

## Abstract

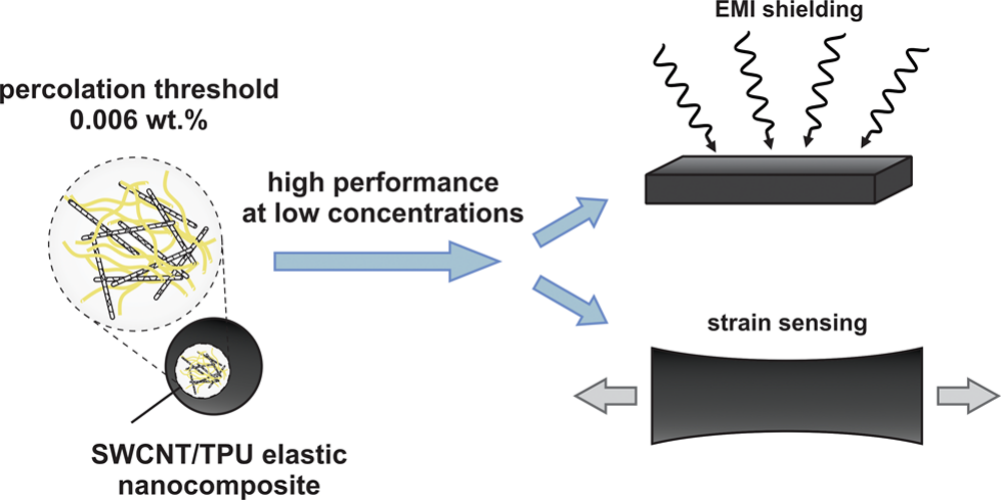

Stretchable and flexible electronics has attracted broad attention over the last years. Nanocomposites based on elastomers and carbon nanotubes are a promising material for soft electronic applications. Despite the fact that single-walled carbon nanotube (SWCNT) based nanocomposites often demonstrate superior properties, the vast majority of the studies were devoted to those based on multiwalled carbon nanotubes (MWCNTs) mainly because of their higher availability and easier processing procedures. Moreover, high weight concentrations of MWCNTs are often required for high performance of the nanocomposites in electronic applications. Inspired by the recent drop in the SWCNT price, we have focused on fabrication of elastic nanocomposites with very low concentrations of SWCNTs to reduce the cost of nanocomposites further. In this work, we use a fast method of coagulation (antisolvent) precipitation to fabricate elastic composites based on thermoplastic polyurethane (TPU) and SWCNTs with a homogeneous distribution of SWCNTs in bulk TPU. Applicability of the approach is confirmed by extra low percolation threshold of 0.006 wt % and, as a consequence, by the state-of-the-art performance of fabricated elastic nanocomposites at very low SWCNT concentrations for strain sensing (gauge factor of 82 at 0.05 wt %) and EMI shielding (efficiency of 30 dB mm^–1^ at 0.01 wt %).

## 1 Introduction

For almost three decades, carbon nanotubes (CNTs) have been the subject of intensive research due to a unique combination of outstanding properties, such as high electrical and thermal conductivity, high specific surface area, aspect ratio, and excellent reinforcement ability.^1^ Meanwhile, nanocomposite materials, especially reinforced polymer matrices, are considered to be one of the largest shares of the nanotube market^2^ as CNT addition to a polymer significantly affects the performance of the material (e.g., improves electrical and thermal conductivity, stiffness, and introduces antistatic properties).^3,4^ The majority of publications on CNT/polymer nanocomposites have been mostly devoted to multiwalled carbon nanotubes (MWCNTs),^3,4^ mainly because of higher dispersibility and substantially lower production costs.^5,6^ Nevertheless, single-walled carbon nanotube (SWCNT)/polymer nanocomposites have been widely reported to be advantageous in comparison to MWCNT-based ones in terms of electrical and thermal conductivity, percolation threshold, mechanical properties, etc.^7−11^ The recent industrial growth of SWCNT production has already changed the balance in SWCNT/MWCNT composites,^2,12^ setting economically eligible prices for SWCNT-based additives for the nanocomposite industry.

Depending on the polymer matrix and the product specifications, CNT/polymer nanocomposite manufacturing usually employs techniques such as solution mixing, melt blending, in situ polymerization, etc.^4,13,14^ In most cases, the data shows a direct relationship between the homogeneity of nanotube dispersion within the matrix to the resultant properties. Despite a higher state of dispersion in the case of in situ polymerization, applicability of this method is often limited to insoluble and thermally unstable polymers.^3,13^ Additionally, the presence of carbon nanotubes has been shown to affect the morphology and structure of various polymer matrices.^14^ At the same time, solution mixing traditionally followed by solvent evaporation often leads to nanotube reagglomeration during the evaporation stage.^3,15^ Nonetheless, adaptation of the solution mixing method known as the coagulation (antisolvent) precipitation technique (CP; aka nonsolvent induced phase separation (NIPS)) maintains the matrix characteristics and opens an avenue for improved spatial dispersion of nanotubes. Proposed by Du et al. in 2003,^16^ the method involves the addition of a poor (anti-) solvent to the CNT/polymer suspension causing immediate polymer precipitation, which results in the polymer chains wrapping around nanotubes. The process results in almost instantaneous composite formation providing relevant insight for the limits of CNT-based composites. Nevertheless, recently, in addition to improvement of spatial distribution homogeneity, many new approaches to fabrication of specifically structured (segregated, layered, porous, etc.) nanocomposites with controlled dispersion of CNTs have been demonstrated to result in low percolation threshold and advanced performance in conductivity-related applications of CNT/polymer nanocomposites,^17−25^ which, however, were beyond the scope of the present study.

Conductive elastomer nanocomposites enhanced by CNTs is a promising field in a variety of soft electronic applications, especially in sensing: strain sensors, pressure sensors, human motion detectors, and others.^25−28^ Such a high interest in mechanical sensing is caused by the gauge factor (ratio of the relative resistance change to the strain), which is usually much higher for nanotube-based nanocomposites than that for traditional strain devices based on metals or semiconductors.^5^ Moreover, such elastic nanocomposites may be utilized as electromagnetic interference (EMI) shielding materials in soft electronics^23,29,30^ as, indeed, typical metal-based materials used for this purpose are not applicable because of their brittleness. Due to elasticity, high affinity to carbon nanomaterials, and wide availability,^31,32^ thermoplastic polyurethane (TPU) is a promising candidate for the fabrication of conductive elastic nanocomposites used in various applications.^31^ However, CNT/TPU nanocomposites rarely employ the coagulation precipitation technique (CP), with little literature existing on the subject, most of which centers around the use of MWCNTs.^6,33,34^ CP provides uniform distribution of CNTs within the TPU matrix, opening prospects in strain sensing applications,^33^ and erosion resistance.^34^ Shin et al.^6^ thoroughly examined CNT/TPU nanocomposites synthesized by NIPS (CP) technique for electrical conductivity, thermal conductivity, mechanical and piezoresistive properties, EMI-shielding efficiency, etc. Moreover, having compared three different types of CNTs, the authors reported superior performance for high aspect ratio MWCNT and SWCNT-based composites for electrical conductivity and related to it EMI-shielding efficiency, while low aspect ratio MWCNTs worked better for piezoresistivity (the authors attributed this fact to a more homogeneous dispersion of shorter MWCNTs). In the referenced papers, the authors used quite high CNT concentrations (up to 10 wt %) and percolation thresholds appeared to be relatively high as well: 0.28 wt % in^33^ and 0.4 wt % in.^6^ This may be related to nonoptimal CNT dispersion techniques preceding the CP process.

In this work, the fast coagulation precipitation technique was utilized for the fabrication of SWCNT/TPU nanocomposites. We employ SWCNTs with moderate length (>5 μm^35^) to reach a golden middle between high aspect ratio and the ease of dispersion procedures, which presumably allows avoiding the problems of an increased tendency to agglomeration and entanglement of SWCNTs with ultrahigh aspect ratio (as was used in ref (6)). Moreover, compared to the previous studies, we have focused on low concentrations of SWCNTs (below 1 wt %) for manufacturing the final nanocomposites. Having combined advantages of fast composite formation with appropriate dispersion techniques, we have managed to achieve superior electrical and mechanical properties of the composites with significantly lower nanotube concentrations. We demonstrated the state-of-the-art performance of the fabricated elastic nanocomposites with low SWCNT concentrations for strain sensing (gauge factor of 82 at 0.05 wt %) and EMI shielding (efficiency of 30 dB mm^–1^ at 0.01 wt %) applications.

## 2 Experimental Section

### 2.1 Raw Materials

SWCNT/TPU nanocomposites were manufactured using commercial elastomeric TPU Ravathane R130A70 in pellet form, provided by Ravago (Turkey). Commercial SWCNT powder, Tuball©, was provided by OCSiAl (Russia). The SWCNTs were characterized at the manufacturer’s end and were shown to have the following dimensions: a length above 5 μm and average diameter of 1.6 nm.^35^

### 2.2 TPU/SWCNT Nanocomposite Fabrication

Initially, TPU granules were vacuum-dried at 80 °C for c.a. 24 h to remove any moisture effect. After drying, TPU granules were dissolved in dimethyl sulfoxide (DMSO, EKOS-1, chemically pure, Russia) with the concentration of 45 g/L. Next, SWCNT powder was added so that the resulting SWCNT/TPU nanocomposites had a specific weight fraction; 0.005, 0.010, 0.025, 0.05, 0.10, 0.25, 0.50, and 1.00 wt %. To improve dispersion homogeneity of the SWCNTs within the polymer matrix, a two-step predispersion procedure was utilized before the coagulation step. First, the SWCNT suspension in the TPU solution was homogenized using a high-speed homogenizer (T-25 digital ULTRA-TURRAX, IKA) for 10 min at 8000 rpm. This was followed by ultrasonication for 45 min using an ultrasonic tip (Branson Sonifier 450) with a power of 200 W. The TPU/DMSO/SWCNT suspension appeared as being black with a uniform consistency and color once the predispersion steps were complete.

**Figure 1:**
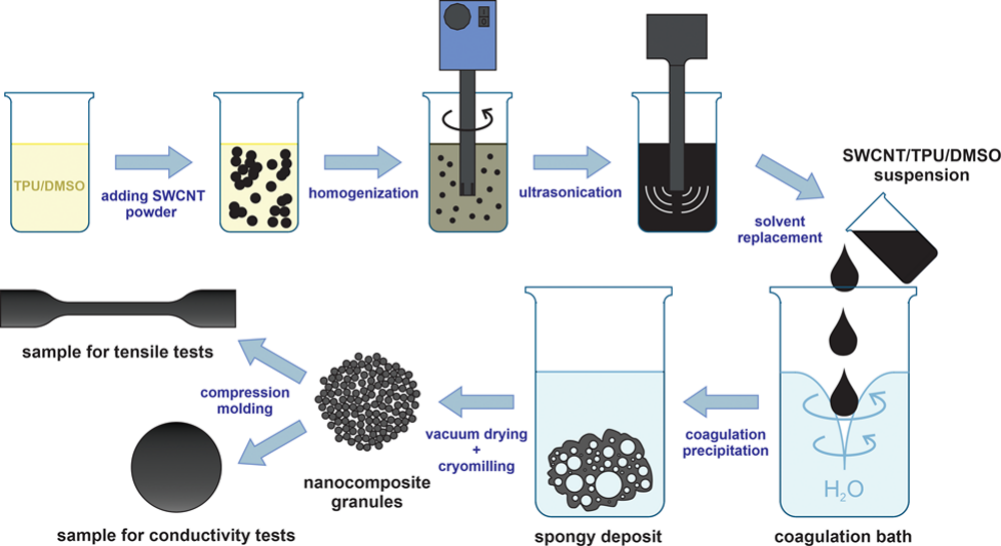
Scheme of the TPU/SWCNT composite fabrication.

Immediately after the sonication, the prepared suspension was poured into a coagulation bath with distilled water under intensive stirring (with a suspension/water volume ratio equal to 1/15) to cause an immediate precipitation. A spongy polymer composite was obtained which was then washed with distilled water to remove any traces of the solvent, vacuum-dried at a temperature of 80 °C overnight, and then frozen using liquid nitrogen for milling with a rotary mill. The color of the nanocomposite powders obtained appeared to range from pale gray to black, depending on the SWCNT content. A similar approach was used by Mazov et al. for the fabrication of MWCNT/polystyrene composites.^36^ After obtaining the nanocomposite powder, it was compression-molded at 180 °C under 2 bar of pressure in a heat-resistant silicone mold for 5 min using a platen hydraulic press (Collin P 300 P/M). Electrical conductivity and EMI-shielding tests utilized disk shaped samples with a thickness of 0.5 mm and diameter of 16 mm, while for tensile and piezoresistivity tests, dumbbell shaped samples with a thickness of 2 mm were used (ISO 37). A schematic representation of the fabrication process is shown in Figure 1. Reference samples of pure TPU were manufactured using the same procedure.

### 2.3 TPU/SWCNT Composite Characterization

#### 2.3.1 SEM Microscopy

Scanning electron microscopy (SEM) was used to investigate the morphology of the nanocomposites using a FEI Teneo scanning electron microscope. Secondary electron mode was used at an accelerating voltage of 10 kV. Fracture surfaces were used to image the microstructure to ensure correlation to testing states. Before imaging, the fracture surfaces were sputtered with gold to enhance charge transfer and improve image quality.

#### 2.3.2 Impedance Spectroscopy

Impedance spectroscopy was used to identify and characterize the percolation behavior of the nanocomposites. Disk shaped samples with a diameter of 16 mm and a thickness of 0.5 mm were used. Impedance measurements were conducted using a coin-cell testing setup in a two-electrode configuration. Impedance spectra were recorded at 200 mV amplitude in the 1 Hz–1 MHz frequency range using a VMP3 Bio-Logic potentiostat/galvanostat. To identify percolation dependencies, the real part of impedance (*Z*_real_) at the lowest frequency (1 Hz) was used. The electrical conductivity (σ_DC_) of the samples was calculated according to the following equation:
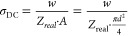
1where *w* is the disk thickness, *A* is the disk contact area, and *d* is the disk diameter.

#### 2.3.3 Tensile Testing and Piezoresistivity Measurements

For compression-molded samples, tensile properties were investigated according to ISO 37 on an Instron 5969 tensile testing machine. Testing was conducted with variable speeds: 5 mm/min traverse speed was used until 4 mm extension for modulus measurement, followed by 500 mm/min traverse speed until specimen failure or maximum traverse travel reached. Strain measurements in the elastic region were performed using a digital image correlation system (Correlated Solutions, U.S., dual 5-megapixel camera setup, VIC-3D software). Elasticity modulus was determined by linear fitting of the stress–strain data gathered from image correlation system within 5% strain range. Larger strains up to 10 mm/mm were calculated based on traverse travel. Limits of the elastic range in the stress–strain curves recorded were calculated by intersecting tangent lines to the initial part of the stress–strain curve and to the second part of the curve corresponding to pronounced plasticity behavior.

For measurements of piezoresistive response, resistance change was monitored simultaneously throughout the tensile tests. The four-point probe scheme were utilized with an initial distance of 2 cm between electrodes. Resistance change was measured using the Keithley 2400 source meter. Strain sensing performance was calculated using gauge factor (GF):
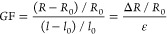
2where *l*_0_ is the initial length (2 cm), *l* is the final length, ε is the strain, *R*_0_ is the initial sample resistance, *R* is the final resistance, and Δ*R* is the resistance change. Two gauge factors (GF) were calculated: the first for the elastic behavior region and the second being the overall GF from start to finish of the tensile tests (elastic + plastic behavior regions).

#### 2.3.4 THz Spectroscopy for EMI-Shielding Efficiency Measurements

EMI-shielding efficiency of the nanocomposites in the THz range was tested using a time-domain spectrometer (TeraView TPS 3000). Both disk shaped samples with a thickness of 0.5 mm (the same were used for impedance tests) and thin film samples were used for testing, ranging in thickness from 0.1–0.2 mm, depending on the SWCNT loading in the nanocomposites. The samples of plane-parallel geometry were fixed on metal holders with a 6 mm aperture. The spectra were recorded in transmission geometry in the range of 4–100 cm^–1^ under vacuum (<0.6 mbar). To compare the shielding efficiency (SE) for the samples with different thicknesses, SE was normalized to the sample thickness (*n*SE) and was calculated as
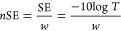
3where *w* is the sample thickness and *T* is the transmittance at a certain frequency.

## 3 Results and Discussion

### 3.1 Structure of SWCNT/TPU Nanocomposites

**Figure 2:**
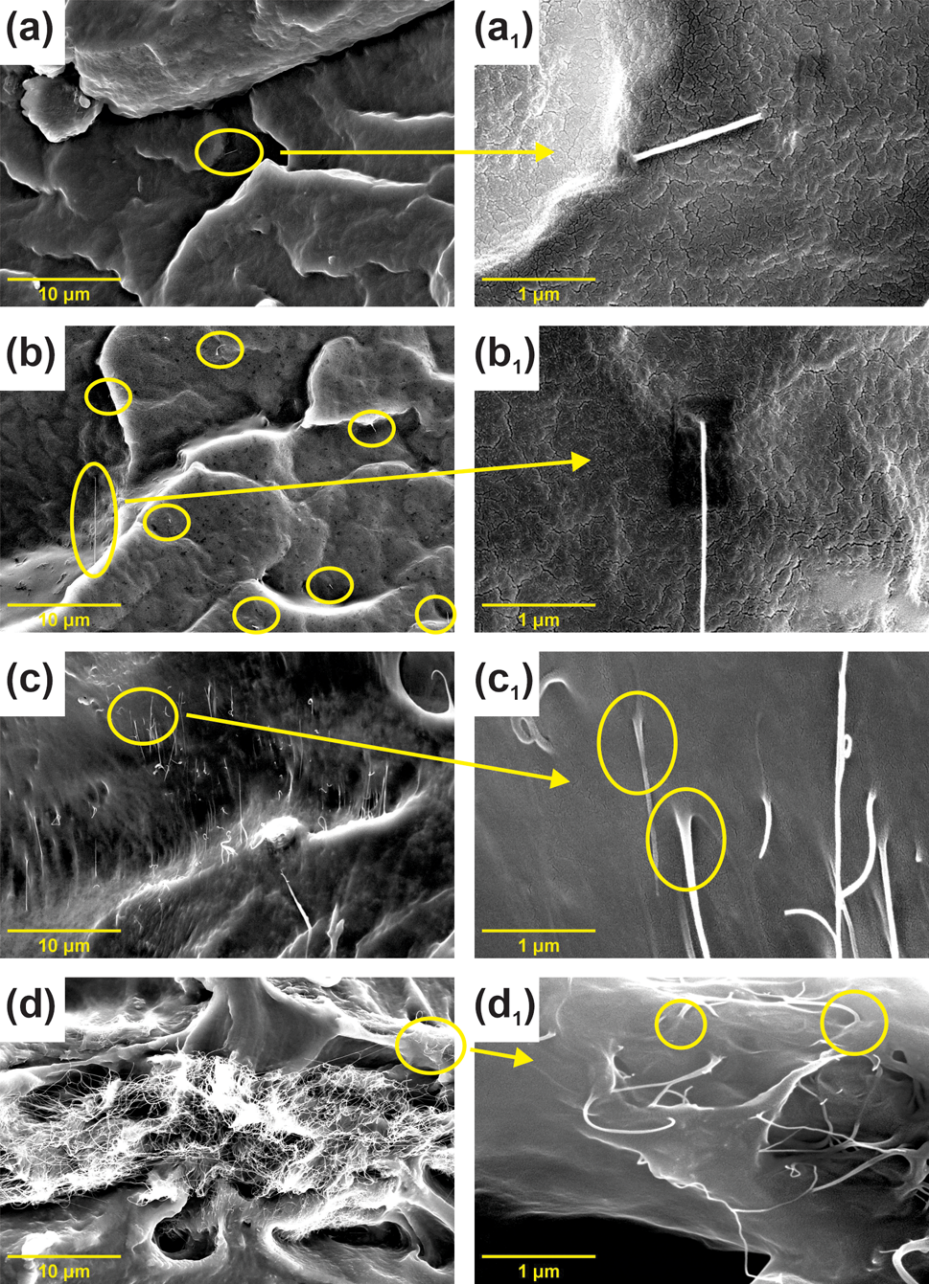
SEM images of the SWCNT/nanocomposite with different SWCNT concentrations (magnification is 10k): (a) 0.005 wt %; (b) 0.05 wt %; (c) 0.25 wt %; (d) 1.00 wt %. a_1_-d_1_) magnified images of the same surfaces (100k). SWCNTs/bundles/aggregates are highlighted with yellow circles; yellow arrows indicated the area on the surface that was taken with a large magnification. On a_1_-d_1_, SWCNT wetted by TPU are highlighted with yellow circles.

The structure and morphology of the nanocomposites were examined with SEM (Figure 2). SWCNTs or their bundles can be clearly distinguished (highlighted in yellow). Evolution of SWCNT dispersion and morphology with the concentration increase can be traced in Figure 2a–d. At the 0.005 wt % concentration (below the percolation threshold), only individual SWCNTs (or their bundles) can be detected (Figure 2a). Nonetheless, for higher SWCNT content, we can estimate SWCNT dispersion in TPU matrix. The SEM image in Figure 2b illustrates quite a homogeneous spatial distribution of SWCNTs/bundles within the polymer at 0.05 wt %. Similar uniform SWCNT distribution can be found on Figure 2c for SWCNT/nanocomposite with 0.25 wt % (yet, SWCNT density obviously increases). Thus, we do not observe tendency for agglomerate formation up to 0.25 wt %. This high dispersion degree can be explained by the effectiveness of the manufacturing route (homogenization + ultrasonication). Furthermore, it also can be caused by the high affinity of TPU macromolecules to nanotube surfaces. Indeed, Figure 2c_1_,d_1_ shows evidently gradual thickening of bundles toward the TPU matrix. It indicates high wetting of nanotubes by TPU which leads to high affinity. It is in a good agreement with the literature data where the high polyurethane affinity to CNT walls was reported by many research groups before.^37,38^ Approaching high concentrations, we observe a clear tendency for the formation of large aggregates of several tens of micrometers (a typical such aggregate is shown on Figure 2c). Nevertheless, the proposed approach was aimed at nanocomposite fabrication with low concentrations. Thus, the morphology displayed concurs with microstructures leading to enhanced mechanical and electrical properties in nanocomposites.^39,40^

### 3.2 Electrical Properties of SWCNT/TPU Nanocomposites

**Figure 3:**
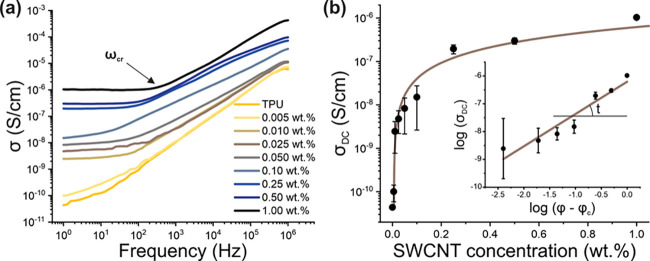
(a) Ac conductivity spectra (Bode charts) of SWCNT/TPU nanocomposites with different SWCNT concentration (indicated in the legend, in wt %); (b) Percolation curve (dc conductivity-vs-concentration dependency) for SWCNT/TPU composites (with calculated fitting function); inset shows the same data plotted in log–log scales for percolation threshold calculation with linear fitting (*R*^2^ is 0.899).

The electrical properties of polymer-based nanocomposites both reflect material applicability and may serve as a method for the evaluation of conductive nanofiller dispersion, aspect ratio, curvature, etc.^5^ For the electrical characterization of CP-prepared SWCNT/TPU nanocomposites, impedance spectroscopy was applied to detail the frequency dependence of the electrical conductivity.^41,42^Figure 3a depicts the impedance spectra (Bode plots) of the SWCNT/TPU nanocomposites. The impedance behavior correlates well with literature data on CNT/polymer composites.^41^ An expected increase in overall conductivity (both dc and ac) with SWCNT concentration was observed. Typically, two conductivity modes are identified, one being frequency-independent at lower frequencies and the second being frequency-dependent at higher frequencies. Increase in conductivity with frequency is usually explained by shorter distances of traveling charge carriers: the charge carriers can avoid high barriers caused by insulating polymers and experience a lower number of intertube junctions.^41^ One more explanation may come out of the expression σ″ = ε_0_ε″ω typical for dielectric materials and describing the response of imaginary part of conductivity to the perturbing wave (which explains the overall impedance increase with frequency valid for pure insulating TPU matrix). Meanwhile, the transition between these two modes corresponding to the so-called “characteristic (critical) frequency” (indicated as “ω_cr_” in Figure 3a) shifts toward lower values for low-content nanocomposites, completely fading at 0.005 wt %. Therefore, in the case of the reference polymer matrix (pure TPU) and SWCNT/TPU nanocomposite with the concentration of 0.005 wt %, frequency dependence typical for dielectric materials is observed,^41^ implying the percolation threshold to be in the range of 0.005–0.010 wt %. The slope of the conductivity-frequency line in the frequency-dependent zone may serve as an indication of the prevailing conductivity mechanism. The conductivity in this region is expressed by a power law:
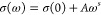
4where σ(0) ≡ σ_DC_ is the dc conductivity, ω is the angular frequency, *A* is the fitting parameter, and *s* is the slope of the line (in log–log scales). The *s* averages in the 0.7–1.0 range, which is a typical value for the tunneling (hopping) conductivity mechanism.^41,43^ A slightly reduced slope seen for the higher SWCNT content nanocomposites may be caused by a lower potential barrier for the tunneling conductivity.

The percolation behavior of CNT/polymer nanocomposites is described by following expression:
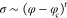
5where σ is the composite conductivity at the lowest frequency applied (considered as dc conductivity, thus, σ ≡ σ_DC_), φ is the SWCNT concentration, φ_*c*_ is the percolation threshold, and *t* is a fitting parameter. Figure 3b represents the concentration dependency of dc conductivity. For the evaluation of φ_*c*_ and *t*, the conductivity-vs-concentration data has been plotted in log–log scales. An optimal φ_c_ value was chosen to provide the best linear fitting of the dependency (inset in Figure 3b).

Analysis of the ac conductivity spectra showed the percolation threshold to be within 0.005–0.010 wt % range. Indeed, the best fitting value has been found at 0.006 wt % (60 ppmw). This value is orders of magnitude lower than what has previously been reported for CNT/TPU nanocomposites, even when orders of magnitude higher aspect ratio SWCNTs have been used with the CP technique^6^ and comparable to the lowest ever reported values of epoxy-based thermosetting nanocomposites where intensive shear mixing techniques were employed.^40,44^ It has appeared to be 1 order of magnitude lower than 0.06 wt % achieved for branched carbon nanostructures (CNS) and reported to be the lowest for TPU-based elastic nanocomposites.^45^ Such a low percolation threshold indicates a high degree of dispersion of the SWCNTs within the polymer and is a result of the optimal combination of dispersion techniques and nanocomposite fabrication method. According to the percolation theory, percolation threshold is determined by the filler aspect ratio:
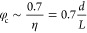
6where η is the filler aspect ratio, *d* is the diameter, and *L* is the length (assuming homogeneous distribution of the filler).^46^ However, even though SWCNTs possess a high aspect ratio, they have a high aggregation/bundling tendency and therefore are difficult to disperse homogeneously, leading to composite underperformance. In this work, SWCNTs with moderate lengths (∼5 μm) are assumed to be easier to disperse with the applied dispersion techniques. With such nanofillers tending to form the conductive network at such low concentration, it can be concluded that the dispersion and fabrication processes used in this study lead to homogeneous spatial distribution of SWCNTs in the TPU matrix. Moreover, the experimentally found percolation threshold (0.006 wt %) has appeared to be noticeably lower the theoretically predicted value for this aspect ratio of ∼3 × 10^3^ (according to eq 6): φ_c_ ∼ 0.035 wt % (assuming SWCNT density as 1.9 g/cm^3^). Such a low value might be associated not with statistical percolation threshold but with so-called kinetic percolation threshold, that is, the case of filler flocculation within the polymer. This segregation is considered to lead to local improvement in intertube contact and formation of the conductive network at much lower concentration. Similar observations were reported^44,47^ and discussed in detail elsewhere.^40^

In classical percolation theory, the value of the slope *t* of the fitted straight line is considered indicative of the conductive nanofiller network dimensionality, which is 2.0 for three-dimensional networks.^5,40^ However, in the majority of studies, the values of *t* are reported lower, reaching values less than 1.0 in some cases.^40^ This may be explained either by the nonuniformity of CNT dispersion, or significant contribution of tunneling conductivity, or reduced dimensionality (possible for thin composite films) or a combination of the stated. The percolation behavior of the studied nanocomposites is not an exclusion, and the fitting line slope *t* is found to be noticeably lower than 2.0: *t* = 1.16 ± 0.16. Taking into account the low percolation threshold and the SEM analysis, significant agglomeration is not possible in this case. On the other hand, owing to the high affinity of TPU to CNTs (observed in the SEM analysis) that leads to nanotube “insulation”, a prevalence of electrical conductivity via tunneling can be the reason behind the reduced *t* value.

The SWCNT/TPU nanocomposites fabricated in this study demonstrate a very low percolation threshold of 0.006 wt % filler concentration. The electrical properties displayed are quite promising and are tied to the optimal SWCNT dispersion and composite fabrication processes.

### 3.3 Mechanical Properties of SWCNT/TPU Composites

**Figure 4:**
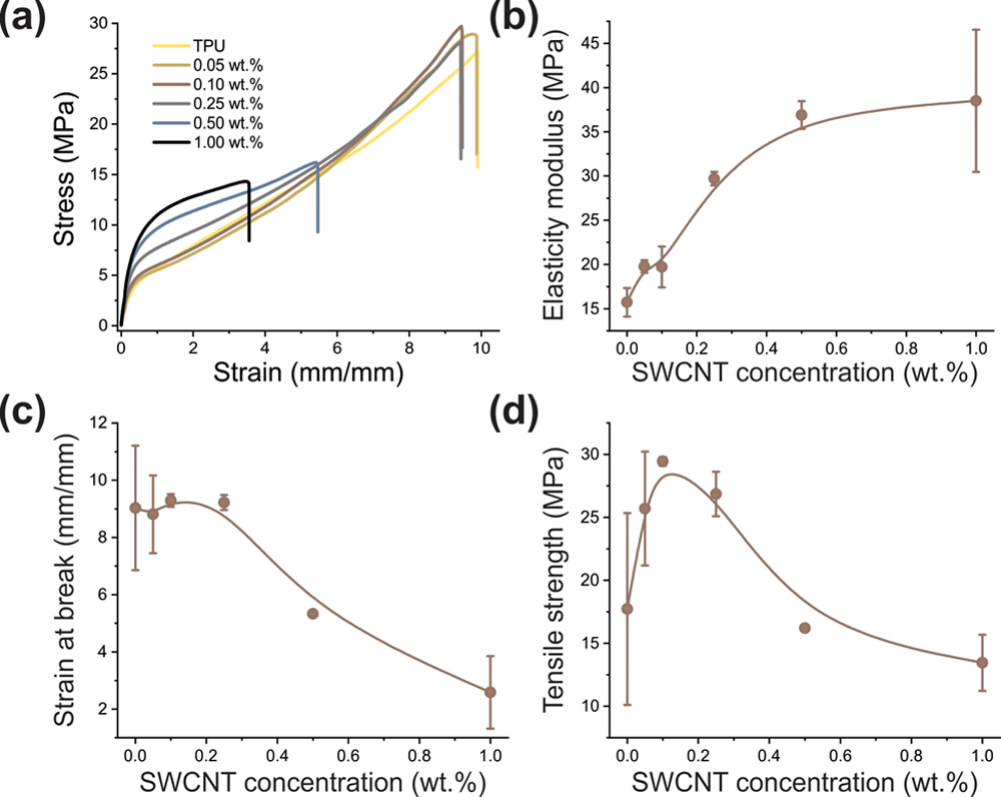
Mechanical properties of SWCNT/TPU nanocomposites: (a) typical stress–strain curves for different concentrations (indicated in the legend); (b) elasticity modulus dependence on SWCNT concentration; (c) strain at break dependence on SWCNT concentration; (d) tensile strength dependence on SWCNT concentration. Error bars represent 1 standard deviation.

The mechanical properties of composites are one of the key factors which influence their practical and industrial applicability. In this study, the tensile properties of the fabricated composites were studied using a tensile testing machine coupled with a digital image correlation system. Figure 4a shows the stress–strain curves for SWNCT/TPU nanocomposites with different nanotube concentrations. Two regimes are clearly observed, an elastic behavior in the low stress region and plastic deformation in the higher. For stretchable electronics, the elastic range is of more concern. Table 1 summarizes the limits of the elastic range for the different nanocomposites. No statistically noticeable shifts in transition strain have been found and, therefore, the addition of SWCNTs to TPU should not limit the operational characteristics of the final nanocomposite.

**Table 1:** Elastic Range and GF (Gauge Factor) Calculated in Elastic Range by Linear Fitting and Maximum GF Found (at the Whole Strain Range)

SWCNT concentration (wt.%)	elastic range (%)	GF in elastic range	maximum GF
TPU	26		
0.05	18	82 ± 2	480 ± 20
0.10	21	35 ± 2	150 ± 10
0.25	24	22 ± 9	1090 ± 30
0.50	21	15 ± 8	780 ± 30
1.00	22	8.0 ± 0.4	15.5 ± 1.8

Figure 4b illustrates an increase in elasticity modulus with an increase in SWCNT content. This concurs with previous polymer matrix nanocomposite research.^15,39^ The increase has a rate of 19.5 GPa to the SWCNT volume fraction (d*Y*/d*V*) slowing down after 0.25 wt %. The total increase is as high as 245% for 1.00 wt % concentration (from 15.7 MPa for neat TPU to 38.5 MPa).

The behavior is comparable or even superior (especially for low nanotube loading, reflected in high d*Y*/d*V* value) to that which has been previously reported concerning polyurethane composites with pristine CNTs (regardless of fabrication method or CNT type),^32,48,49^ including CP based studies.^6,34^ Certainly, covalently modified CNTs possess stronger interaction with polymers leading to better stress transfer to CNTs and, hence, higher modulus values,^39,50^ which is beyond the present tasks nonetheless. We consider the mechanical performance properties revealed to be due to the homogeneous spatial distribution of the nanotubes with high aspect ratio (∼10^3^). These factors were also found to be decisive in the previous studies.^39,51^ One more possible reason for the modulus increase is nanotube-induced crystallization of soft segments in TPU macromolecules occurring through a heterogeneous nucleation mechanism.^52^ Yet, such a detailed analysis of evolution of mechanical properties deserves a separate study.

The strain at break (Figure 4c) demonstrates an interesting behavior: it remains constant until 0.25 wt % then it drops sharply, indicating changes in plasticity. Such a dependence together with gradual growth of the nanocomposite stiffness with SWCNT content results in the pattern of tensile strength versus nanotube concentration (Figure 4d), with a pronounced maximum at 0.10 wt %. An increase in elastic stiffness and a corresponding decrease in plasticity with higher SWCNT loading leads to failure occurring at lower elongations and, correspondingly, lower stresses. Such brittleness may be caused by enhanced agglomeration at high nanotube content,^31^ which prevents TPU penetration into the percolation network and acts as local stress concentration points,^32^ which is in good agreement with the SEM analysis of SWCNT/TPU nanocomposite at 1.00 wt % concentration (Figure 2d). This assumption may be strengthened by the fact that elastic modulus growth for higher SWCNT contents decreases, implying insufficient interfacial stress transfer (between TPU and SWCNTs). Nevertheless, the proposed approach was aimed to low nanotube concentrations and, indeed, it has appeared to be beneficial for low SWCNT loadings up to 0.25 wt %, where significantly increased elasticity modulus and tensile strength are found at relatively constant elongation at break value.

### 3.4 Piezoresistivity of SWCNT/TPU Nanocomposites

**Figure 5:**
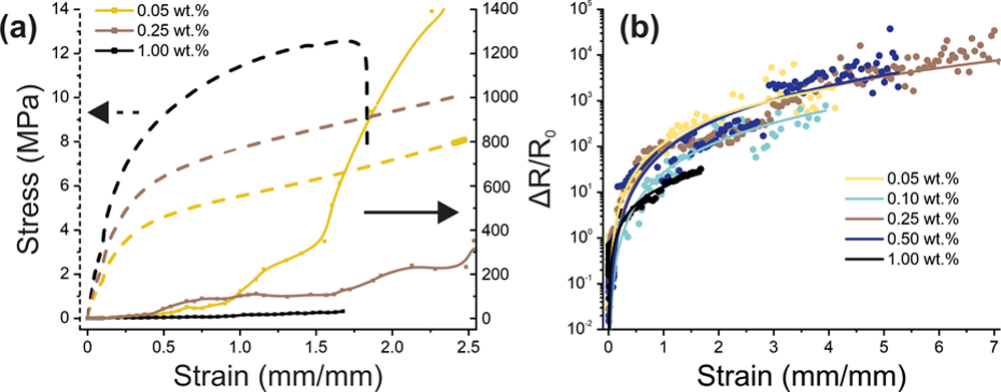
(a) Normalized resistance change on strain dependency (right *y*-axis) plotted with stress–strain curves (left *y*-axis) for the SWCNT/TPU nanocomposites with the concentrations of 0.05, 0.25, and 1.00 wt %. (b) Normalized resistance change in strain dependency within the full strain range for all the concentrations of SWCNT/TPU nanocomposites studied for piezoresistivity with the fitting function (linear fitting in log–log scales was used).

One of the most promising features of conductive elastic nanocomposites is piezoresistive response to mechanical loading, which allows them serving as a functional material in sensing applications for soft electronics. Figure 5a represents the pattern of normalized resistance change (Δ*R*/*R*_0_) with the strain applied for different nanocomposites (0.05, 0.25, and 1.00 wt %) plotted together with the corresponding stress–strain curves, while Figure 5b depicts the same dependencies within the full strain range.

We note significant deviations in the piezoresistive behavior between nanocomposites with different SWCNT concentrations (Figure 5a). While for high-concentration nanocomposites, the piezoresistive response is linear, composites with lower SWCNT content demonstrate exponential growth of the resistance change. For small strains within the elastic range, the response is rather linear for all the samples. This response expressed in gauge factor (GF) has been estimated by linear fitting the data in the elastic range and is presented in Table 1. Consistent decline in strain sensitivity with nanotube concentration is evident. The highest GF calculated has an extremely high value of 82 and corresponds to the SWCNT concentration of 0.05 wt %. The reported values for CNT/PU nanocomposites vary considerably and depend on CNT type and aspect ratio,^5^ as well as composite structure type (layered, fibrous, etc.).^24,25,32,53^ CNT-based nanocomposites with high aspect ratio (leading to a stronger percolation network and thus less susceptible to physical changes; often, the case of SWCNT type) are usually reported as lower strain-sensitive; therefore, it may be said that the nanocomposites display high piezoresistive response caused exactly by good dispersion.^5,10^ Accurate measurement of piezoresistive responses at lower loading (expected to be higher as closer to the percolation threshold) are hindered due to the high resistivity of the low concentration nanocomposites, thereby, sample resistance reaches values of a few GΩ immediately with stretching, which is beyond the measurement limits.

The observed piezoresistivity trends agree with percolation theory and previous studies.^5^ Three major forms of percolation changes contribute to this piezoresistive response: deformation of the entire percolation network due to loading (1), disruption of physical connections between nanotubes (2), and changes in tunneling distances between CNTs (due to exponential dependency of resistance on the distance between the nanotubes and CNT network reorganization) (3).^5^ For highly developed percolation networks of highly concentrated composites, increasing applied strain causes deformation and displacement of the network (1), which does not lead to noticeable resistance drop because of an abundance of conductive paths still existing. Thus, linear response exists while there are still a sufficient number of nanotube interconnections. Subsequent transition to higher response occurs when disconnection of conductive fillers becomes unavoidable (2). Meanwhile, inherent exponential growth of the resistance is seen to be determined by the tunneling mechanism of CNT/polymer nanocomposites (3).

At extremely high strains, the composite essentially turns into a dielectric, where no considerable resistivity changes follow. Curiously enough, maximum GFs (maximum value at the whole strain range) found for each composite vary considerably (Table 1). Meanwhile, the highest sensitivity of the middle-concentrated nanocomposites has been revealed. It is caused by a combination of the factors: general higher sensitivity for lower concentrations, initial resistance and composite ductility. Although lower-filler concentration containing nanocomposites are more sensitive at the low strains (in elastic range), their resistance reaches values of GΩs rapidly, indicating their dielectric nature and further strain does not lead to noticeable changes in behavior. On the contrary, at high filler concentration, the composites are both low-sensitive and brittle. However, within medium filler concentrations, the composites have relatively low resistance in the beginning of tensile loads and are ductile. Figure 5b depicts the trends discussed above. Exclusively because the fact that, for high strains (above 200–300%), middle-concentrated nanocomposites (0.25 and 0.50 wt %) are still conductive and, hence, their resistance is measurable, the sensitivity reaches the highest values. However, the data are noticeably noisy at high strains, therefore, we used a fitting function (constructed in log–log scales) for the GF estimation.

Nonetheless, for stretchable electronics, GF in elastic range is of higher interest. Thus, the approach to SWCNT/TPU nanocomposite fabrication proposed in this work may be considered promising and beneficial for mechanical sensing applications since a combination of very low percolation threshold and good mechanical properties achieved by high dispersion degree facilitates high piezoresistivity at relatively low concentrations (of 0.05 wt %).

### 3.5 EMI-Shielding in THz Range

Another demanding application of conductive elastic nanocomposite is electromagnetic interference (EMI) shielding in soft electronics. In particular, EMI shielding in THz range is required for medical diagnostics, imaging, wireless communications, transistor technologies, characterization of materials, and many others.^54−58^ Carbon nanomaterials are of high interest because of their lightweight and lower fabrication cost compared to conventional quasi-optical devices.^58^ Despite the little research devoted to THz spectroscopy of CNT/polymer nanocomposites, available articles state that the nanocomposites demonstrate high EMI-shielding efficiency in this range.^59−61^

**Figure 6:**
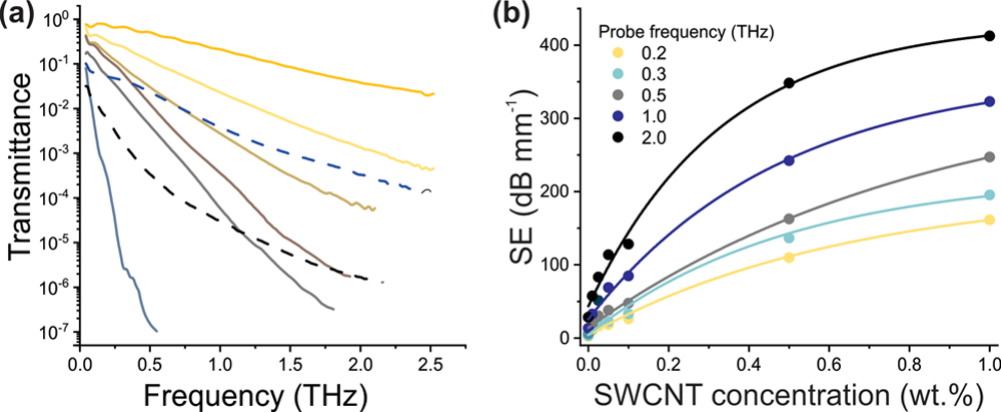
(a) THz transmittance spectra of SWCNT/TPU composites with different concentrations; the thickness of the plane-parallel samples is 0.5 mm (unless otherwise stated); (b) SE normalized to the thickness of the sample as a function of concentration for several fixed frequencies.

The SWCNT/TPU nanocomposites were tested for EMI-shielding efficiency in the terahertz range using time-domain THz spectroscopic measurements conducted in transmission geometry. Figure 6a represents transmittance spectra in the 0.1–2.5 THz range. We observe the steady drop of transmittance with SWCNT concentration and frequency. In works by Gorshunov et al. and Zhukova et al.,^62,63^ the Drude conductivity model (typical for metals) was successfully applied for the explanation of SWCNT network electrodynamic response based on terahertz-infrared spectroscopy measurements. According the model, metal-like conductivity of SWCNT network within TPU matrix can be considered responsible for such a low transmittance of the composites. Due to transmission constraints, samples of a lower thickness were manufactured (indicated in the legend in brackets in Figure 6a; for all the other samples, thickness is 0.5 mm).

Shielding efficiency (SE) normalized to the thickness (in dB mm^–1^) was calculated from transmittance spectra according to eq 3. SE is considered as a total result of the three effects: reflection from the sample, absorption within the sample and multiple reflection between sample plane faces (the Fabry–Perot effect). Since multiple reflection is characteristic for low-absorbing materials, its influence for CNT-based nanocomposites is negligible and therefore shielding is determined by absorption and reflection.^64^Figure 6b illustrates SE dependence for selected probe frequencies on SWCNT concentrations. SE grows with higher frequencies and SWCNT content. This correlates well with previously reported results on THz SE for CNT/polymer composites.^59,60^ On the other hand, the values of SE obtained for SWCNT/TPU nanocomposites fabricated by the present method have appeared to be significantly higher: 20 dB efficiency (requirement for the devices operating in X-band) at 1 THz for 1 mm thick composite film can be reached even for very low concentration of 0.01 wt.%. This may be explained by the fact that unlike pure SWCNT films, nanocomposite interaction with incident EM fields depends not only on SWCNT conductivity but on the quality of nanotube dispersion as well, which influences nanocomposite conductivity. Thus, highly homogeneous dispersion of nanotubes within TPU in this study has a positive influence on SE. Curiously, unlike the work by Polley et al.,^60^ we do not observe a linear increase in SE with SWCNT concentration: the growth is the sharpest for the lowest nanotube content and increase in concentration shows a reduced growth. Such a trend is in good agreement with percolation behavior since far over the percolation limit, a large number of conductive paths are already present and not leading to crucial growth in conductivity and, hence, SE. Otherwise, just above the percolation threshold, the conductive pathways contribution is crucial in both, conductivity and associated SE. Thus, this finding indicates on eligibility of the current approach as high SE efficiency is achieved at very low nanotube concentrations.

### 3.6 Comparison with Existing Literature

To show the applicability of the proposed method, we need to compare the properties and performance of the elastic nanocomposites fabricated in this study to those of the similar nanotube-based nanocomposites demonstrated in the literature. Table 2 represents the lowest percolation threshold values found for the CNT/TPU elastic nanocomposites with different types of CNTs and produced by different fabrication techniques.

**Table 2:** Percolation Threshold Values Achieved in the Papers Related to CNT/TPU Nanocomposites (Sorted by the Publication Year)

filler type	aspect ratio	processing	percolation threshold (wt %)	ref
MWCNT	∼100	solution mixing	0.28	(52)
MWCNT	∼160	solution mixing	0.35	(65)
MWCNT	∼160	melt mixing	0.13	(66)
carbon nanostructure (CNS)	70 μm length; 20 μm thickness	melt mixing	0.06	(45)
MWCNT	∼160	melt mixing	3.02	(32)
MWCNT	∼160	fused filament fabrication +3D printing	2–3	(67)
MWCNT	∼10^3^	CP (NIPS)	1.5	(6)
MWCNT	∼10^4^	CP (NIPS)	0.7	(6)
SWCNT	10^5^ – 10^6^	CP (NIPS)	0.4	(6)
SWCNT	∼10^3^	CP (NIPS)	0.006	this work

Comparison with the recent works reveals at least 1 order of magnitude drop in the percolation threshold values achieved in this study, which verifies the proposed approach.

**Figure 7:**
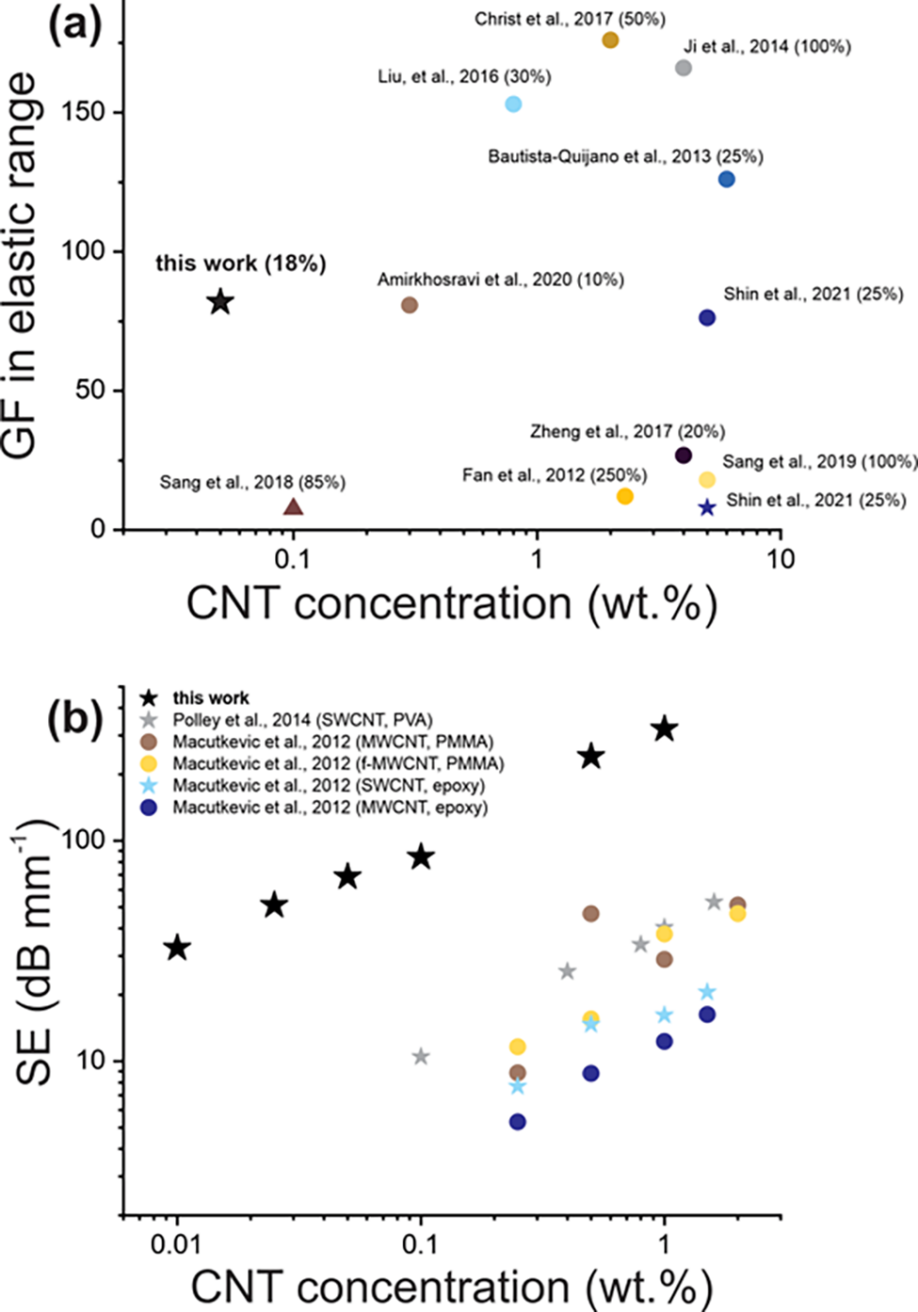
(a) The current advances in the gauge factor values shown versus CNT concentration^6,22,24,32,33,45,68−71^ (strain shown in the brackets); ★ corresponds to SWCNTs, ● to MWCNTs,▲ to carbon nanostructures (CNS) based on MWCNTs. (b) The current advances in the values of SE at 1 THz normalized to the sample thickness (types of CNTs and polymer matrix are shown in the brackets).^59−61^

Despite some works stating extremely high sensitivity of TPU-based nanocomposites (expressed in GF of several thousand^45^), performance estimated in the elastic range (before plasticity region is reached) is more relevant since it is only in this range that the nanocomposite-based mechanical sensor can be used in continuous mode (indeed, for cycling tests, strain usually does not exceed 100%). Thus, Figure 7a demonstrates GF values calculated from the reported literature and showing the piezoresistive response within the elastic range (strain values are given in the brackets).

The data point showing a GF of 82 achieved in this work at 0.05 wt % is obviously located to the left of the other points implying better comparative performance in strain sensing at 1–2 orders of magnitude lower weight concentrations. General trend of the sensitivity decline with the filler concentration represented in Table 1 and discussed in the literature^5^ may indicate insufficient dispersion degree of the CNTs resulted in high GF at high CNT concentrations shown in Figure 7a.

Figure 7b illustrates SE in THz range (at 1 THz) of the CNT/polymer nanocomposites versus CNT weight concentrations achieved in the recent works. Although the intuitive tendency of increased shielding at higher nanotube concentrations can be observed in each referred work, SE values demonstrated in this study exceed these by 1 order of magnitude, which emphasizes the prospects of the current method of elastic nanocomposite fabrication.

## Conclusions

Conductive elastic nanocomposites based on carbon nanotubes are of high interest because of their functionality and applicability in soft/portable electronics. Taking into account general prerequisites for supremacy of SWCNT-based elastic nanocomposites (compared to MWCNT-based ones) we examined the utilization of SWCNT/TPU-based elastic nanocomposites with small nanotube concentrations. For this, we employed the coagulation precipitation technique (nonsolvent induced phase separation). The obtained high dispersion degree of the SWCNTs within the polymer was ensured by combination of homogenization andultrasonic treatment with instantaneous precipitation of the nanocomposite. The selected fabrication route has resulted in almost uniform spatial distribution of nanotubes within the matrix, yielding a percolation threshold as low as 0.006 wt % (60 ppmw). The homogeneity of the distribution resulted in the properties of elastic nanocomposites superior to that reported so far; namely, for mechanical properties (245% growth of elasticity modulus for 1.00 wt % SWCNT), piezoresistive performance (gauge factor (GF) in the elastic range is of 82 for 0.05 wt %) and electromagnetic interference (EMI) shielding efficiency in THz range (30 dB mm^–1^ at 1 THz at 0.01 wt %). Thus, functional elastic nanocomposites produced by the method described in this work may be promising candidates for soft electronic applications. We believe the obtained results will encourage academia and industry to search for new solutions/applications of this method.
